# Expression Profiling of Flavonoid Biosynthesis Genes and Secondary Metabolites Accumulation in *Populus* under Drought Stress

**DOI:** 10.3390/molecules26185546

**Published:** 2021-09-13

**Authors:** Umair Ahmed, Muhammad Junaid Rao, Cheng Qi, Qi Xie, Hamza Armghan Noushahi, Muhammad Yaseen, Xueping Shi, Bo Zheng

**Affiliations:** 1 Key Laboratory of Horticultural Plant Biology of Ministry of Education, College of Horticulture and Forestry Sciences, Huazhong Agricultural University, Wuhan 430070, China; umairhzau@outlook.com (U.A.); ChengQi_4039@163.com (C.Q.); xieqi19931013@163.com (Q.X.); 2 State Key Laboratory for Conservation and Utilization of Subtropical Agro-Bioresources, College of Agriculture, Guangxi University, Nanning 530004, China; mjunaidrao@webmail.hzau.edu.cn; 3 College of Plant Science and Technology, Huazhong Agricultural University, Wuhan 430070, China; hamzanaushahi143@gmail.com; 4 Wuzhishan National Long-Term Forest Ecosystem Monitoring Research Station, Hainan Key Laboratory for Sustainable Utilization of Tropical Bioresource, College of Forestry, Hainan University, Haikou 570228, China; shyaseen521@gmail.com

**Keywords:** *Populus*, drought stress, flavonoid biosynthesis pathway, gene expression, secondary metabolites

## Abstract

Flavonoids are key secondary metabolites that are biologically active and perform diverse functions in plants such as stress defense against abiotic and biotic stress. In addition to its importance, no comprehensive information has been available about the secondary metabolic response of *Populus* tree, especially the genes that encode key enzymes involved in flavonoid biosynthesis under drought stress. In this study, the quantitative real-time polymerase chain reaction (qRT-PCR) analysis revealed that the expression of flavonoid biosynthesis genes (*PtPAL*, *Pt4-CL*, *PtCHS*, *PtFLS-1*, *PtF3H*, *PtDFR*, and *PtANS*) gradually increased in the leaves of hybrid poplar (*P*. *tremula* × *P*. *alba*), corresponding to the drought stress duration. In addition, the activity and capacity of antioxidants have also increased, which is positively correlated with the increment of phenolic, flavonoid, anthocyanin, and carotenoid compounds under drought stress. As the drought stress prolonged, the level of reactive oxygen species such as hydrogen peroxide (H_2_O_2_) and singlet oxygen (O_2_^−^) too increased. The concentration of phytohormone salicylic acid (SA) also increased significantly in the stressed poplar leaves. Our research concluded that drought stress significantly induced the expression of flavonoid biosynthesis genes in hybrid poplar plants and enhanced the accumulation of phenolic and flavonoid compounds with resilient antioxidant activity.

## 1. Introduction

**Figure 1:**
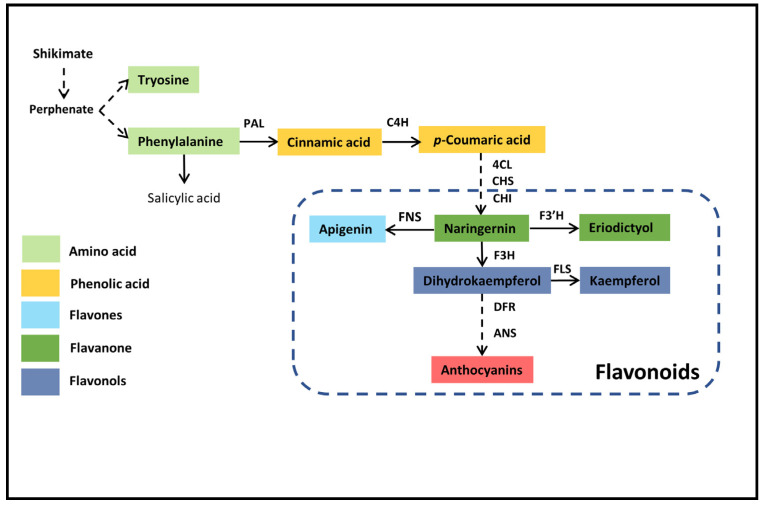
Illustration of the biosynthetic pathways leading to the major groups of phenolic compounds in *Populus*. [22].

The plant growth and survival are greatly affected by water availability and different environmental constraints [1]. Globally, water shortages have caused huge economic losses to the agriculture and forestry sectors. Among various abiotic stresses, drought stress plays an important role in restraining plant growth and productivity [2,3,4,5] by affecting the biochemical and physiological attributes of the plant [6,7,8]. Plants adapt various strategies to cope with drought stress and oxidative damage, including maximizing the use of water, minimizing loss of water [9,10], developing antioxidant systems [11,12], and various biochemical, morphological, and physiological drought-resistance mechanisms to compensate for water lose [13,14]. Drought resistance is a complicated trait that is regularized by different genes, associations between genes and environmental signals, involving many morphological and metabolic pathways [15]. The genes that respond to drought stress encode proteins that perform diverse functions such as signal transduction, gene expression, control for stress damage, and remedies [16]. The phenylpropanoid metabolic pathway produces various secondary metabolites, which possess resilient antioxidant activity under abiotic stress conditions. Plant phenolic compounds, especially flavonoids, are very powerful compounds in plants that can provide resistance to a variety of biotic and abiotic stresses [17,18,19,20]. Flavonoids belong to the phenolic group and consist of two aromatic rings, which are jointed by three carbons. Although their functions and structures are diverse, flavonoids are usually derived through the phenylpropane pathway (secondary metabolic pathway). In this pathway, the chalcone synthase is the entry point, catalyzing the conversion of malonyl-CoA and 4-coumaroy CoA to chalcone, thereby initiating the biosynthesis of flavonoids [21]. The major enzymes entangled in the formation of various flavonoids include chalcone isomerase (CHI), flavanone 3-hydroxylase (F3H), dihydroflavonol 4-reductase (DFR), flavonol synthase (FSH), and eventually the anthocyanins synthase (ANS) (Figure 1). 

Phenolic and flavonoid contents are affected by various abiotic and biotic stresses, and among various species and tissues, their response is different [8,23,24,25]. The *Populus trichocarpa* enhanced the flavonoid contents when exposed to UV-B radiation [26]. In the leaves and roots of pistachio (*Pistacia vera* L.), phenolic contents elevated in response to drought stress. Khoyerdi and Gharibi [27,28] reported the increment of total flavonoid and phenolic contents under water stress in *Achillea* species. The up-regulation of secondary metabolites constitutes the basis for plants to adapt and evolve to environmental changes under different stress conditions [29]. The information obtained from the up-regulation of secondary metabolites in plants will help to develop stress-resistant plants in the future.

The *Populus* genus is one of the most cultivated tree genera due to its multiple uses for the timber, bioenergy, and paper industries. It consists of six subgenera: Tacamahaca, Turanga, Aigeiros, Leucoides, Leuce, and Abaso. The trees from this genus grow at temperate latitudes and are mostly fast growing [30]. The availability of a high-quality and well-annotated *P*. *trichocarpa* genome sequence has allowed us to develop molecular tools to investigate whole transcriptome of *Populus* [31,32]. These developments provide us ample opportunities to study transcriptome-based responses to drought stress in tree species and substantial variations inspected inside genus *Populus* both in terms of biomass accumulation and survival [11,33,34]. For tree research, *Populus* has become a model plant [35]. With its whole genome sequenced, *P. trichocarpa* is an important resource for genomic and genetic research [32]. Meanwhile, the hybrid poplar *P. tremula* × *P. alba* INRA no. 717–1B4 (hereafter referred to as poplar 717), due to its efficiency and ease of genetic transformation and in vitro regeneration, is widely used in molecular biology research [36]. Plants’ secondary metabolites play a key role by detoxifying the reactive oxygen species during biotic and abiotic stresses, as a previous study revealed that increased flavonoids content enhanced pathogen resistance in *Populus* [37]. A similar study showed the increased metabolite profiling of *Populus* under pathogen stress [38]. Drought stress severely affects the growth and development of plants, and species of *Populus* have different responses to drought stress. In this study, poplar 717 was investigated to unveil its response to drought stress, the expression of genes related to flavonoid biosynthesis, and the response of secondary metabolites to drought stress. 

## 2. Results

### 2.1. Morphological Changes in Poplar Leaves under Drought Stress

**Figure 2:**
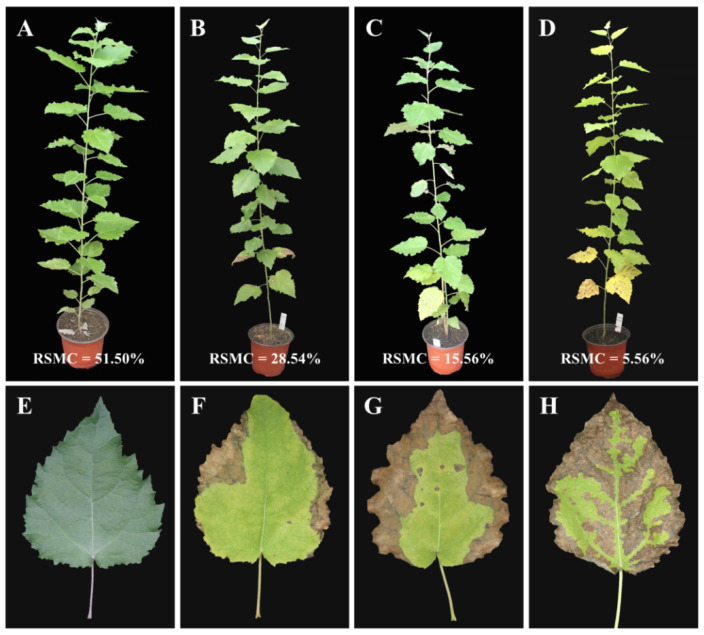
Morphological changes of basal leaves in poplar 717 under different relative soil moisture contents (RSMC). (**A**) D0 plant, (**B**) D5 plant, (**C**) D10 plant, (**D**) D15 plant, (**E**) D0 basal leaf, (**F**) D5 basal leaf, (**G**) D10 basal leaf, (**H**) D15 basal leaf.

The drought stress was applied at three different time points, 5 days, 10 days, and 15 days (D5, D10, and D15), while 0-day (D0) plants were considered as control. The relative soil moisture content (RSMC) at D0 was 51.50%, which gradually decreased upon longer stress, and in D5, D10, and D15 plants, it dropped down to 28.94, 15.56, and 5.56%, respectively (Figure 2). The D0 leaves were normal and showed no symptoms of drought stress. On D5, one to two leaves at the basal stem started to wilt, and the symptoms appeared on leaf margins, while more leaves started to wilt at D10 and D15 and the stress symptoms spread to the whole leaf, with some leaves turning yellow due to stress severity.

### 2.2. Photosynthesis under Drought Stress in Poplar

**Figure 3:**
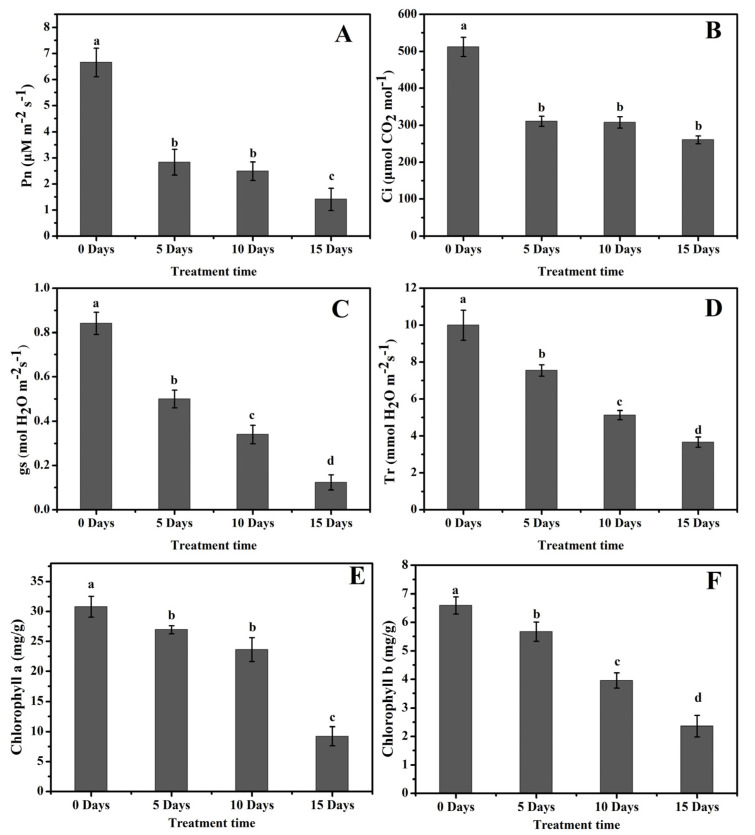
Photosynthetic attributes of poplar leaves under drought stress. (**A**) Net photosynthetic rate (Pn), (**B**) Intercellular CO_2_ (Ci), (**C**) Stomatal conductance (gs), (**D**) Transpiration rate (Tr), (**E**) Chlorophyll a, (**F**) Chlorophyll b. The values are presented as means ± standard error (SE) (*n* = 3). The different small letters indicate a significant difference (*p* < 0.05) among different treatments.

The photosynthetic changes in poplar leaves under drought stress were investigated by considering different photosynthetic parameters, including net photosynthetic rate (Pn), stomatal conductance (gs), intercellular CO_2_ (Ci), and transpiration rate (Tr) (Figure 3). The results revealed that Pn decreased from 6.5 µM m^−2^ s^−1^ in D0 plants to 2.5, 2.0, and 1.0 µM m^−2^ s^−1^ in D5, D10, and D15 plants, respectively (Figure 3A). Pn constantly decreased as the drought stress prolonged (*p* ≤ 0.001) (Table 1). Ci also decreased with drought stress progression, which recorded two folds lower in drought-treated plants in comparison to control (Figure 3B). Additionally, gs and Tr also significantly decreased in poplar leaves as the drought stress duration increased (*p* ≤ 0.001) (Table 1). The gs was 0.9 mol H_2_O m^−2^ s^−1^ in control plants, which was two to three folds higher than in drought-treated plants (Figure 3C), and a similar trend was observed in Tr as well, which dropped down from 10 mmol H_2_O m^−2^ s^−1^ in D0 to 6.5, 4.5, and 3.0 mmol H_2_O m^−2^ s^−1^ in D5, D10, and D15 plants, respectively (Figure 3D). Chl a and Chl b decreased as drought increased. Under control conditions, Chl a and Chl b were 31 and 6.5 mg/g, respectively (Figure 3E–F). The lowest Chl a and Chl b values were observed on D15, which were 9.0 and 2.5 mg/g respectively, about three-folds lower than those of D0 plants. Chl a and Chl b values were 27 and 5.6 mg/g on D5 and 24 and 4.0 mg/g on D10, respectively. This indicates a gradual decrease in both Chl a and Chl b with the increase in drought stress.

### 2.3. Expression Profiling of Flavonoid Biosynthesis Genes under Drought Stress in Poplar Leaves

**Figure 4:**
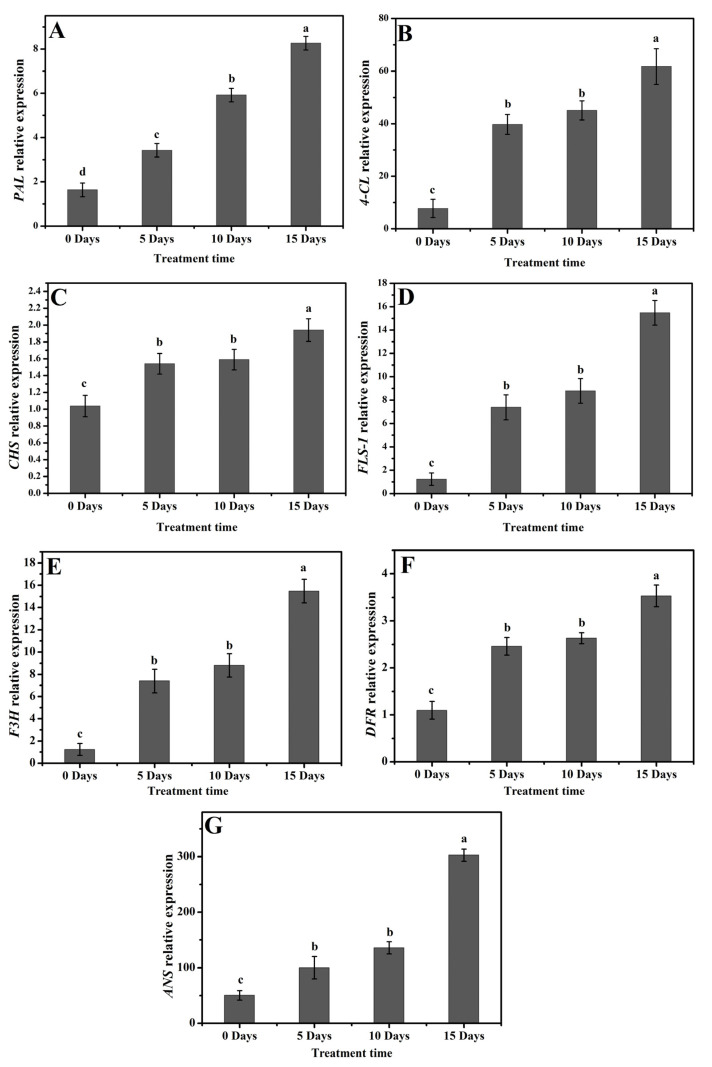
Relative expression of genes involved in the flavonoid biosynthesis pathway under drought stress in poplar 717 leaves. (**A**) *PAL*, phenylalanine ammonia-lyase, (**B**) *4-CL*, 4-coumarate CoA ligase, (**C**) *CHS*, chalcone synthase, (**D**) *FLS-1*, flavonol synthase, (**E**) *F3H*, flavanone 3-hydroxylase, (**F**) *DFR*, dihydroflavonol-4-reductase, (**G**) *ANS*, anthocyanidin synthase. The gene expression values are presented as means ± standard error (SE) (*n* = 3). The different small letters indicate a significant difference (*p* < 0.05) among different treatments.

The expression profiling of flavonoid biosynthesis genes under drought stress in poplar 717 was investigated by qRT-PCR. The early unbranched part of the flavonoid biosynthesis pathway is encoded by *PAL*, *CHS,* and *CHI* genes. A significant increase in *PAL* was observed under drought stress in contrast to control plants, and the expression gradually increased upon longer stress (Figure 4A). In D0 plants, the expression level was low, but on D15, the expression level was four times more than the D0 plants. A relatively higher expression level of the *4CL* gene was noted under drought stress, and the maximum gene expression was observed on D15 (three times higher than D0), and its expression level on D5 and D10 (about 2.5 times higher than D0) showed a relative increasing pattern in comparison to control plants (Figure 4B). The *CHS* gene expressed steadily and had the maximum expression on D15. Compared with all stress-treated plants, its expression level in D0 plants was relatively low (Figure 4C). The biosynthesis of flavonoids in the later stages is encoded by *FLS-1*, *F3H*, *DFR*, and *ANS* genes. The *FLS-1* and *F3H* genes also exhibited higher expression. The expression of these two genes increased significantly, and as the duration of the stress increased, the expression continued to increase, and the maximum was recorded on D15 (Figure 4D,E). Additionally, under drought stress, the expression levels of *DFR* and *ANS* were much higher. Both genes in the D15 plants were three to four times higher than those in the D0 plants (Figure 4F,G). All these results indicate that the application of drought stress significantly increased the expression of flavonoid biosynthesis genes. The relative expression levels of these genes increased with the progress of stress.

### 2.4. Accumulation of Total Phenolics, Flavonoids, and Carotenoids Content in Poplar Leaves under Drought Stress

**Figure 5:**
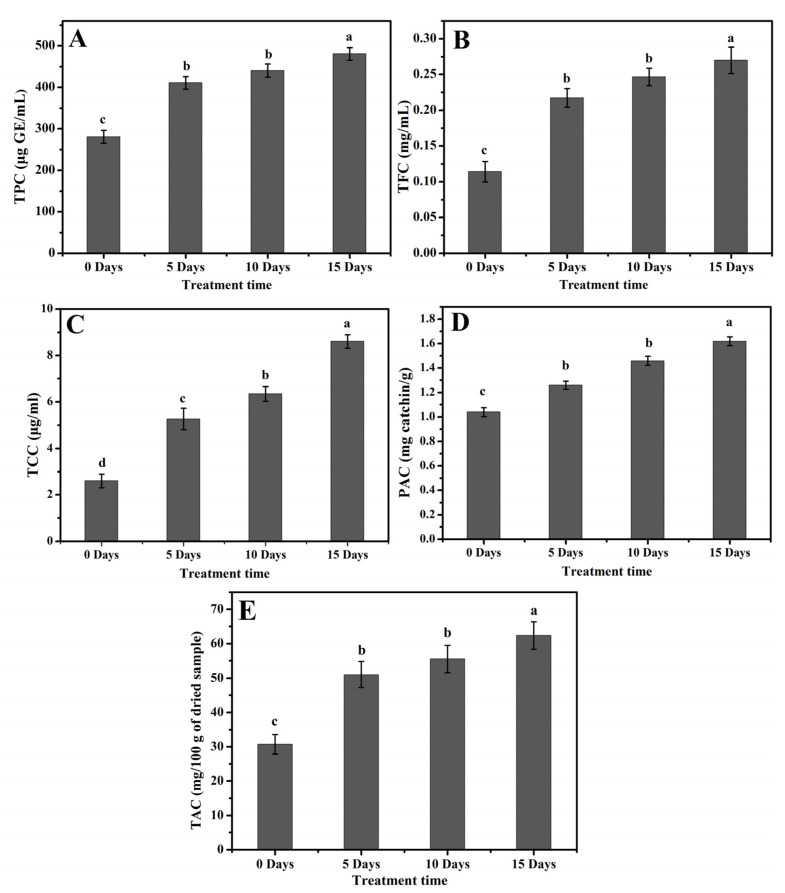
Accumulation of secondary metabolites in *Populus* leaves under various drought stress treatments. (**A**) Total phenolic contents (TPC), (**B**) Total flavonoid contents (TFC), (**C**) Total carotenoid contents (TCC), (**D**) Proanthocyanidin contents (PAC), (**E**) Total anthocyanin contents (TAC). The data are presented as fold change relative to the control (samples at 0 days of treatment). The values are presented as means ± standard error (SE) (*n* = 3). The different small letters indicate a significant difference (*p* < 0.05) among different treatments.

The content of total phenolics, flavonoids, and carotenoids (TPC, TFC, and TCC, respectively) produced a prolific response to drought stress in poplar 717 (*p* ≤ 0.001) (Figure 5A–C) (Table 2). TPC showed a gradual increase on D5 (400 µg GE/mL) and D10 (420 µg GE/mL) in comparison to control (280 µg GE/mL), while a significant increase in TPC was recorded on D15 (490 µg GE/mL) (Figure 5A). Compared with the lowest value of 0.10 mg/mL in D0 plants, TFC increased in D5, D10, and D15 plants, with the values of 0.20, 0.25, and 0.30 mg/mL, respectively (Figure 5B). TCC also increased steadily under drought stress, and its values in D0, D5, D10, and D15 plants were 2.5, 4.5, 6.0, and 9.0 µg/mL, respectively (Figure 5C).

### 2.5. Accumulation of PAC and TAC in Poplar Leaves under Drought Stress

The plants under drought stress showed an enhanced level of PAC, as shown in Figure 5D. The D0 plants showed relatively lower contents of PAC, whereas the highest PAC was recorded on D15 (1.6 mg catechin/g). On D5 and D10, the PAC values were 1.2 and 1.4 mg catechin/g, respectively (Figure 5D). A similar pattern was observed while evaluating TAC (*p* ≤ 0.001) (Table 2). The highest value of 65 mg/100 g dried samples were recorded on D15. The values recorded on D0, D5, and D10 were 30, 50, and 55 mg/100 g of dried samples, respectively (Figure 5E).

### 2.6. Enhanced Antioxidant Capacity and Antioxidant Activity 

**Figure 6:**
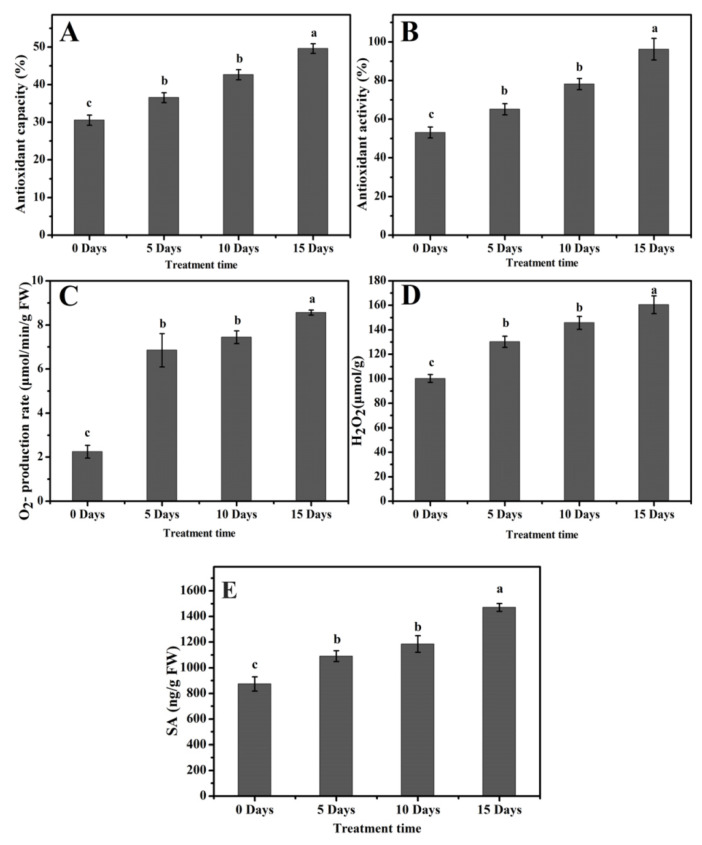
Antioxidant, reactive oxygen species, and salicylic acid response to drought stress in *Populus* leaves under drought stress. (**A**) Antioxidant capacity, (**B**) Antioxidant activity, (**C**) O_2_^–^ production rate, (**D**) H_2_O_2_ production, (**E**) Salicylic acid. The values are presented as means ± standard error (SE) (*n* = 3). The different small letters indicate a significant difference (*p* < 0.05) among different treatments.

The antioxidant capacity and activity were enhanced under drought stress (*p* ≤ 0.001) (Table 2). With the increase in drought stress time, the antioxidant capacity in poplar 717 plants was greatly enhanced. The antioxidant capacity of D5, D10, and D15 plants were 35%, 40%, and 50%, respectively, which were higher than that of D0 plants (30%) (Figure 6A). The value of leaf antioxidant activity of poplar trees under drought stress also increased significantly. D0 plants had the lowest antioxidant activity value, and D15 plants had the highest value, which was 46% and 96%, respectively (Figure 6B). 

### 2.7. High Accumulation of O_2_^−^, H_2_O_2_, and Salicylic Acid under Drought Stress

A higher O_2_^−^, H_2_O_2_, and salicylic acid production was recorded in poplar leaves to drought stress (*p* ≤ 0.001) (Table 2). Compared with D0 plants, significant increases in O_2_^−^ levels were observed on D5, D10, and D15. The concentration in D0 plants was 2.3 µM min^−1^ g^−1^ FW, while the values after drought stress increased to 7.0, 7.5, and 8.8 µM min^−1^ g^−1^ FW, respectively (Figure 6C). In addition, the D0 plants accumulated a relatively lower level of H_2_O_2_ at 95 µM/g. A steady increase in H_2_O_2_ content was observed on D5, D10, and D15, which were 125, 135, and 160 µM/g, respectively (Figure 6D).

The salicylic acid (SA) actively responded under drought stress, and its production increased as the stress duration prolonged (*p* ≤ 0.001) (Table 2). In the leaves of D0 plant, its content was 820 ng/g FW; in D5, D10, and D15 plants, the content increased to 1050, 1200, and 1430 ng/g FW, respectively (Figure 6E).

### 2.8. Correlation Analysis

**Figure 7:**
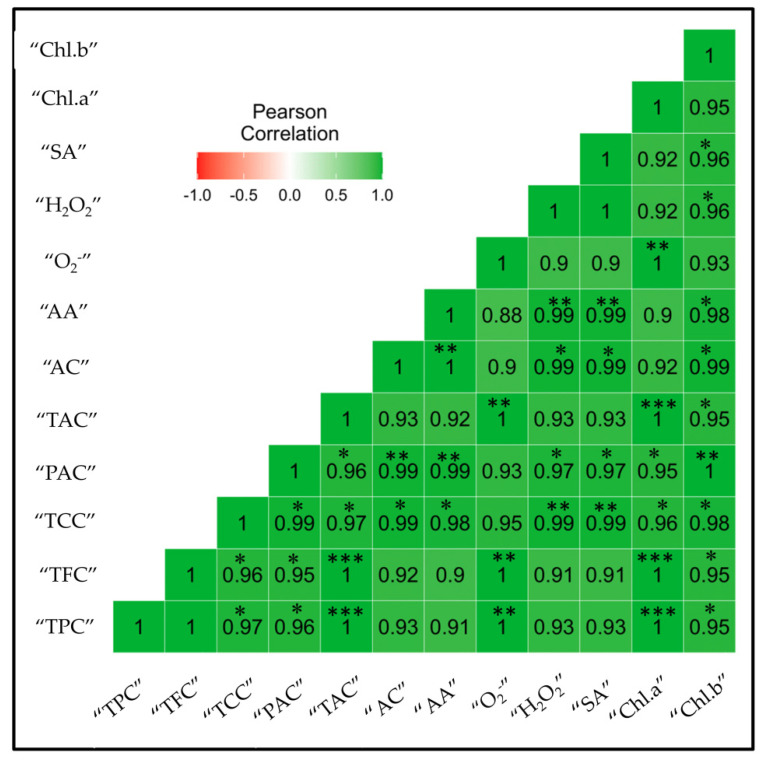
Correlational matrix of secondary metabolic compounds in poplar 717 leaves under drought stress. The colored gradient legends represent coefficients of correlation r-values from +1.0 (dark green) to −1.0 (dark red). Significant effects are indicated in boldface as follows: *** *p* ≤ 0.001, ** *p* ≤ 0.01, and * *p* ≤ 0.05. All coefficients were computed by the Pearson correlation for possible pairs of variables in the matrix.

Pearson’s correlation analysis among different variables is represented in Figure 7. The results unveiled that TPC had a positive highly significant correlation (*** *p* ≤ 0.001) with TAC, Chl a, O_2_^−^, and TAC (Figure 4). TFC also showed highly positive results (*** *p* ≤ 0.001) with Chl a and TAC. TCC revealed a positive significant correlation (** *p* ≤ 0.01) with H_2_O_2_ and SA. Additionally, PAC also showed a positive significant correlation with Chl b and antioxidant capacity. TAC revealed a positive highly significant correlation (*** *p* ≤ 0.001) with Chl a and positive moderate significant correlation (** *p* ≤ 0.01) with O_2_^−^. Moreover, antioxidant activity also had a positive moderate significant correlation (** *p* ≤ 0.01) with H_2_O_2_ and SA. Overall, the correlation heat map indicated that the correlation matrix among secondary metabolic and various biochemical compounds revealed a positive correlation under the drought stress conditions in poplar. The correlation analysis among flavonoid biosynthesis genes and total phenolic and flavonoids content is represented in the supplementary file (Figure S1). The results revealed that all flavonoid biosynthesis genes were positively correlated with total phenolics and flavonoids content.

## 3. Discussion

Plant phenolic compounds, especially flavonoids, are very powerful compounds in plants that can provide resistance to a variety of biotic and abiotic stresses. There are different classes of metabolites; among them, flavonoids due to their tremendous antioxidant activity are most prominent. Phenolic and flavonoid contents are affected by various abiotic and biotic stresses, and among various species and tissues, their response is different. Due to the biotic and abiotic stress in plants, ROS production increases. The flavonoids are non-enzymatic antioxidants, which minimize the harmful impacts of ROS in plants [39]. Photosynthesis is sensitive to ROS accumulation in plants because most of the photosynthetic enzymes are preferred targets for oxidation. In the leaves of *Amaranthus tricolor*, drought stress induced a significant reduction in photosynthetic pigments such as chlorophyll a and chlorophyll b contents and also decreased photosynthetic apparatus efficiency [40]. In this experiment, the drought-treated plants resulted in decreased various photosynthetic parameters such as Tr, gs Ci, Tr, Chl a, and Chl b (Figure 3). Yu [41] observed a similar pattern of decreased photosynthetic parameters in hybrid poplar *P. simonii* × *P. nigra* under H_2_O_2_ stress. Complex responses at cellular, developmental, and physiological levels are initiated by drought stress in plants. Flavonoid gene expression and metabolism are directly affected by drought stress [42]. One of the renowned secondary metabolic pathways under stress conditions in plants is the flavonoid biosynthesis pathway, in which many genes evolve in response to stress [43,44]. The entry point of this pathway is the *CHS* gene, while CHI catalyzes the conversion of chalcone to flavanone, which is subsequentially converted into many other flavonoids. Under intense drought stress, higher *CHS* expression was reported In our study, a similar expression pattern of the *PtCHS* gene was observed. As the stress duration increased, the expression of *PtCHS* increased and reached the maximum level in D15 plants (Figure 4C). In response to higher *PtCHS* expression levels, higher TFC was observed on D15 (Figure 5B). The activity of *PAL* gene arouses distinct secondary metabolite groups, for example, the phytohormone salicylic acid [45]. Several channels are involved in the biosynthesis of salicylic acid. PAL and isochorismate synthase are involved in one of the channels—the Shikimate pathway [46]. An increase in *PAL* gene expression enhances salicylic acid production [45]. These reports comply with our results. A significant increase in salicylic acid production (Figure 6E) was noted with the increased *PtPAL* gene expression (Figure 4A). The maximum *PtPAL* gene expression was detected on D15, and at the same time, the maximum salicylic acid production was recorded in D15 poplar plants. 

Another key enzyme in the biosynthesis of flavonoids is F3H, which plays a crucial role under abiotic and biotic stress. Studies conducted in *Reaumuria soongorica* [47] and grape berries [48] showed higher *F3H* transcripts under drought stress. In our study, the induction of *PtF3H* was quite significant under intense drought stress (Figure 4E), which possibly indicates that higher *PtF3H* expression may induce drought tolerance in poplar plants. *FLS-1*, *DFR*, and *ANS* are considered as the downstream genes in the flavonoid biosynthetic pathway. Research conducted on *P. euramericana* indicates that higher abiotic stress increased the expression of these genes [49]. In our study, the expression of *PtFLS-1*, *PtDFR*, and *PtANS* were remarkably higher at the maximum stress point. All these genes were expressed in a similar expression pattern: their expression increased with the progress of stress (Figure 4D,F,G).

Under adverse environmental conditions, modifications in plant flavonoid biosynthesis occur to cope with the situation [46]. Plants with the higher flavonoid concentration can better deal with oxidative stress, which may be due to their higher flavonoid antioxidant potential [50]. Our results also indicate that under drought stress, the biosynthesis level of flavonoids in poplar plants was higher, and the expression level of genes related to flavonoids biosynthesis was higher, too (Figure 4). 

The plant phenolic compounds, similar to some amino acids and derivatives of phenylprepanoid, have tremendous antioxidant potential; these compounds help plants deal with unfavorable environmental conditions [51]. TPC comprehensively increased in two wheat cultivars, Chinese Spring and Aikang 58, under drought stress [2]. In this study, higher TPC was found under drought stress, and the TPC production increased with the stress duration (Figure 5A). Higher antioxidant capacity and activity help plants mitigate the negative effects of drought stress by reducing ROS production. The plants with higher antioxidant activity or capacity show more resistance against drought stress [52], and those with lower antioxidant activity or capacity are susceptible to drought stress [53]. Prolonged exposure to drought and high light stress enhanced antioxidant activity and capacity in citrus plants [54,55]. In our experiments, increased exposure to drought stress of poplar plants also increased their antioxidant capacity and activity (Figure 6A−B). The production of H_2_O_2_ is triggered by drought stress, which damages the cellular components and causes protein oxidation. A higher level of H_2_O_2_ causes redox imbalance in plants during a progressive drought period [53]. This also complies with our results; a progressive drought period induced higher H_2_O_2_ in poplar plants (Figure 6C).

The tremendous antioxidant ability has been shown by anthocyanins that assist plants in reducing ROS damages. In the flavonoid biosynthesis pathway, anthocyanin is the end product that possibly can be the reason for higher anthocyanin production after drought stress. The massive high accumulation of flavonoids leads to the rapid biosynthesis of anthocyanin [56]. These are consistent with our results. Under drought stress, the total anthocyanin biosynthesis of poplar plants increased (Figure 5E). Free radicals are oxidative and strongly reactive, which in cells harm DNA and proteins [57,58]. In context of this, many non-enzymatic antioxidants e.g., flavonoids and phenolics, have greater antioxidant potential. The antioxidant characteristics of these compounds are primarily due to their tendency for scavenging oxidizing elements that are involved in the production of free radicals [59,60]. The flavonoids and phenolics compounds may also reduce harmful environmental impacts; hence, these compounds are synthesized under stress conditions in plants such as drought stress to minimize the negative impacts of oxidative stress [61]. These are in compliance with our results. The TPC and TFC are positively correlated with superoxide radicle (O_2_^−^); this indicates that as the O_2_^−^ production under progressive drought stress increases, the TPC and TFC also increase (Figure 7). The metabolism and biosynthesis of carotenoids is highly affected by drought stress. Carotenoids are important antioxidant pigments that play a crucial rule in plant resistance against stress conditions; these pigments maintain redox balance by eliminating free radicals and ROS production [62,63]. The TCC in our study also responded well under progressive drought stress (Figure 5A).

## 4. Materials and Methods

### 4.1. Plant Material and Growth Conditions

Poplar 717 plants were tissue cultured in 250 mL plastic bottles, containing 35 mL 1/2 Murashige and Skoog (MS) medium (Phytotech, Lenexa, KS, USA) with 0.6% (*w/v*) agar and 2% (*w/v*) sucrose. Then, the plants were placed in a growth chamber with a photoperiod of 16 h of light and 8 h of dark at 25 °C for three months (January to March 2020). Subsequently, the plants were transplanted into pots (8.5 cm in diameter and 14 cm in height), filled with soil, and grown in an open greenhouse at a temperature of 28 ± 3 °C. The ratio of peat, matrix, and vermiculite in the soil mixture was 3:2:1. The plants were grown in the soil for four months (April to August 2020) followed by imposing drought stress. 

### 4.2. Drought Stress Treatment

To apply the drought stress, a batch of soil-grown poplar 717 (105–110 cm in height) was divided into four groups, each with six plants. The plants were grown under a 16/8 h light/dark photoperiod, with a light intensity of 250–300 µmol photo m^−2^ s^−1^, 70% relative humidity, and at a temperature of 25 ± 3 °C. Three groups of plants were not watered for 5 days (D5), 10 days (D10), and 15 days (D15) to obtain mild drought, moderate drought, and severe drought, respectively. The plants in the control group (D0) were watered regularly as per their evaporative demand. The relative soil moisture content was measured by using a soil moisture meter (Field Scout^TM^ TDR 300, Spectrum Technologies, Inc., Aurora, IL, USA). The leaf samples of the 9th node were taken with three biological replicates, and immediately placed in liquid nitrogen and then stored at −80 °C for further analysis. 

### 4.3. Measurement of Photosynthetic Parameters

The net photosynthetic rate (Pn), stomatal conductance (gs), intercellular CO_2_ (Ci), and transpiration rate (Tr) in poplar leaves were measured by using a portable photosynthesis system (LICOR 6400 XT, LI-COR Inc., Lincoln, NE, USA) [64]. These photosynthetic parameters were measured at three points (upper, middle, and bottom leaves).

For chlorophyll a and chlorophyll b content analysis, the poplar leaf tissues (500 mg) were ground and homogenized in 10 mL 80% acetone solution [65]. The incubation of the homogenized mixture was done for 4 h in dark at room temperature and then centrifuged at 12,000× *g* for 5 min. For the evaluation of chlorophyll a and chlorophyll b contents (Chl a and Chl b, respectively), the supernatant was spooled out, and a spectrophotometer was used to investigate the absorbance of chlorophyll a and b at 645 and 663 nm, respectively. The formula used to measure Chl a and Chl b is as follows:

Chl a (mg/L) = A_663_ × 12.7 − A_645_ × 2.69 Chl b (mg/L) = A_645_ × 22.9 − A_663_ × 4.68 (1)

### 4.4. Total RNA Extraction cDNA Synthesis and qRT-PCR

The RNA was extracted from the leaves (approximately 70 mg) of the poplar plant by using the Ultrapure RNA Purification Kit (CoWin Biosciences, Boston, MA, USA) as per the manufacturer’s instructions. The quality and quantity of RNA were analyzed on a NanoDrop^TM^ 2000 (Thermo Scientific, Waltham, MA, USA), followed by running on 2% (*w/v*) agarose gel electrophoresis. The cDNA was synthesized by using a PrimeScript^TM^ RT Reagent Kit with gDNA Eraser (TaKaRa, Dalian, China) with a reaction volume of 20 µL according to the manufacturer’s instructions. The cDNA was diluted (20×) with deionized water (dH_2_O) and used as a template for PCR amplification. ChamQ^TM^ SYBR^®^ qPCR Master Mix with High ROX Premixed (Vazyme, Nanjing, China) was utilized in quantitative real-time polymerase chain reactions (qRT-PCR) by following the standard protocol of manufacturer’s instructions. The qRT-PCR was performed by using white 384-well plates on a Light Cycler^®^ 480 instrument II (Roche, Wilmington, MA, USA). The 2^−ΔΔCt^ method was applied to obtain the relative gene expression value [66]. *PtActin* was used as the reference gene for qRT-PCR [67]. The primers used in this experiment are listed (Supplementary Table S1).

### 4.5. Extraction and Evaluation of Secondary Metabolites from Poplar Leaves

#### 4.5.1. Total Flavonoids Content and Total Phenolics Content

To evaluate the total flavonoids content (TFC) and total phenolics content (TPC) in the leaves of poplar 717 after drought stress, leaf samples (100 mg each) were ground and homogenized in 5 mL of 80% methanol. The samples were incubated on an orbital shaker at a rotation speed of 200 rpm at room temperature for 2 h and then centrifuged at 8000× *g* for 5 min. The supernatant was transferred into a new tube, and the pellet went through the extraction steps one more time. Then, the supernatants were combined for the measurement of TFC and TPC [68]. For TFC estimation, 0.5 mL supernatant was mixed with 2.25 mL dH_2_O and 0.15 mL 5% sodium nitrite solution (NaNO_2_) and was incubated at room temperature for 5 min. Then, 0.3 mL 10% aluminum chloride hexahydrate (AlCl_3_·6H_2_O) solution was mixed into the reaction solution prepared above and incubated for 5 min, which was followed by the addition of 1 mL 1 M sodium hydroxide (NaOH) solution and vortexed for 1 min.

Folin–Ciocalteu reagent (FCR) was used to estimate TPC content as reported [68]. The 10× diluted FCR in a quantity of 2.25 mL was mixed with 0.3 mL of the methanolic extract made above in a 10 mL tube, which was incubated for 6 min at room temperature. Then, 2.25 mL 6% sodium carbonate (Na_2_CO_3_) was added in the reaction solution and incubated for 2 h at room temperature. The standard curve for TFC and TPC was generated by using rutin equivalents (RE) and gallic acid equivalents (GAE), respectively. TFC (mg RE/g) and TPC (mg GAE/g) of fresh leaf samples were evaluated by measuring the absorbance at the wavelength of 530 nm and 725 nm on a UV-1800 spectrophotometer (Shimadzu, Tokyo, Japan), respectively [69].

#### 4.5.2. Total Carotenoids Content

The total carotenoids content (TCC) was evaluated by grinding and homogenizing 100 mg of poplar leaves in 1 mL of 80% methanol solution [65], which was followed by centrifugation at 4000× *g* for 10 min. The supernatant was transferred into a new tube, and the extraction was repeated once. Both supernatants were combined in one tube for evaluation of TCC. The concentration of carotenoids was determined by measuring the absorbance at the wavelength of 470 nm on the spectrophotometer, and the following formula was used

TCC (μg/mL) = (1000 × A_470_ − 1.63 × Chl a − 104.96 × Chl b)/221 (2)

#### 4.5.3. Proanthocyanidins and Total Anthocyanin Content

The content of proanthocyanidins (PAC) was estimated as described previously [70], with minor modifications. Poplar leaves (30 mg) were ground in liquid nitrogen, and then, 1 mL of extraction solution (70% acetone, 29.5% water, and 0.5% acetic acid) was added. The standard curve for PAC estimation was generated by using a catechin compound, and PAC values are expressed in mg catechin/g leaf sample. The PAC was determined by measuring the absorbance at the wavelength of 550 nm.

The total anthocyanin content (TAC) was determined as previously described [71]. The leaf tissue (100 mg) was homogenized in an extraction solution mixture containing 45% methanol (*v/v*) and 5% acetic acid (*v/v*), which was followed by centrifugation at 10,000× *g* for 10 min at room temperature. The spectrophotometer was used to measure the absorbance of total anthocyanin contents at 530 nm and 657 nm. The formula used to measure TAC is the following:

TAC (mg/100 g of DW) = A_530_ − (0.25 × A_657_) × 5 times of extraction volume (mL) × 1/dry weight of leaf sample (g) (3)

### 4.6. Investigation of Antioxidants Capacity, Antioxidants Activity, and H_2_O_2_ and O_2_^−^ Production under Drought Stress

For estimation of antioxidants capacity and antioxidants activity, 100 mg of leaf tissue was homogenized in 1 mL of extraction mixture (1% acetic acid, 29% distilled H_2_O, and 70% ethanol). The centrifugation was done at 8000× *g*, and 30 µL supernatant was taken and mixed with 2.97 mL 0.1 mM 2, 2-diphenyl-1-picrylhydrazyl (DPPH). The incubation was done in the dark for 30 min at room temperature. The reaction mixture without a sample was taken as the control. The antioxidant capacity and antioxidant activity were estimated by using the spectrophotometer to measure the absorbance at 517 nm [72]. The standard curve for antioxidant capacity and antioxidant activity was created by using Trolox equivalent, and antioxidant capacity values are given in mM trolox/100 mg leaf sample. 

The following formula was used for measuring antioxidant (free radical scavenging) activity:

Antioxidant activity (%) = 1 − (A_517_ sample/A_517_ control) × 100% (4)

For the evaluation of H_2_O_2_, 100 mg of poplar leaves were homogenized in 1 mL of 1% trichloroacetic acid using an ice bath and then centrifuged at 10,000× *g* for 10 min [73]. The spectrophotometer was used to measure the hydrogen peroxide (µM/g) of the dried leaf samples at 390 nm. The estimation of superoxide radical (O_2_^−^) was done by taking 100 mg of poplar leaf tissues [74], whereas reactive oxygen species (ROS) were evaluated with the help of a Fluorometric Assay Kit (CAT # E-BC-K138-F, Elabscience, TEX, USA) as per following the producer’s instructions with minor modifications. The fresh leaf tissues (100 mg) were taken for O_2_^−^ analysis. The O_2_^−^ unit was estimated as a 0.1 unit change in absorbance/min at corresponding wavelength values.

### 4.7. Estimation and Evaluation of Salicylic Acid

The salicylic acid was measured by modifying the previous method [75]: placing 100 mg of homogenized poplar leaf tissues in 1 mL of dH_2_O. After centrifugation, we transferred 500 µL of the supernatant into a new tube with 2.5 mL of freshly prepared 0.1% ferric chloride. An iron complex is made after the reaction of ferric acid with aqueous salicylic acid, which gives a violet color. The spectrophotometer was used to measure the absorbance at 540 nm.

### 4.8. Statistical Analysis

The statistical software Statistix 8.1 (Analytical Software, Inc., Tallahassee, FL, USA) was used in this research work for data evaluation. Excel (Microsoft Corp., Redmond, WA, USA) was used for finding the standard error and mean values. One-way ANOVA analysis was used to evaluate the effect of drought stress in poplar leaves. Significant differences among different treatments were found by multiple comparisons with the LSD test at significant difference *p* < 0.05 by using Statistix 8.1 software. The graphs were generated by using OriginPro 8.5.1 (OriginLab Corporation, Northampton, MA, USA).

## 5. Conclusions

Based on the results of our study, we have concluded that antioxidants including flavonoids are very important secondary metabolites for *Populus* plants to cope with harsh environmental conditions. We analyzed the production of secondary metabolites and the expression of related genes in poplar trees under drought stress. When the plants were under drought stress, the expression of genes related to the production of secondary metabolites increased, including *PtPAL*, *PtCHS*, *PtCHI, PtFLS-1*, *PtF3H*, *PtDFR*, and *PtANS*. Ultimately, the higher expression of these genes leads to a higher production of various secondary metabolites including flavonoids, phenolics, carotenoids, proanthocyanidins, and anthocyanins. More insights into the function of these genes under drought stress in *Populus* are required, but this study will certainly be useful and provide comprehensive insight into flavonoids (and other secondary metabolites) activity in response to drought stress in hybrid poplar plants.

## Figures and Tables

**Table 1:** Influence of drought stress on photosynthetic parameters in poplar 717 leaves.

Photosythetic Parameters	*F* Ratio	*p*-Value
Pn	83.07	0.001 ***
Ci	83.07	0.001 ***
gs	133.37	0.001 ***
Tr	227.71	0.001 ***
Chl a	107.39	0.001 ***
Chl b	9.14	0.0058

Notes: Pn: mean net photosynthetic rate; Ci: intercellular CO_2_; gs: stomatal conductance; Tr: transpiration rate; Chl a: chlorophyll a; Chl b: chlorophyll b. Degree of freedom (Df) = 3. Significant effect is indicated in boldface as follows: *** *p* ≤ 0.001.

**Table 2:** Impact of drought stress on secondary metabolic compounds in poplar 717 leaves.

Secondary Metabolites	*F* Ratio	*p*-Value
TPC	146.45	0.001 ***
TFC	106.74	0.001 ***
TCC	153.74	0.001 ***
PAC	202.61	0.001 ***
TAC	308.46	0.001 ***
AC	240.60	0.001 ***
AA	85.82	0.001 ***
O_2_^-^	122.85	0.001 ***
H_2_O_2_	150.40	0.001 ***
SA	257.54	0.001 ***

Notes: TPC: total phenolic contents; TFC: total flavonoid contents; TCC: total carotenoid contents; PAC: proanthocyanidin; TAC: total anthocyanin contents; AC: antioxidant capacity; AA: antioxidant activity; O_2_^–^: superoxide radical; H_2_O_2_: hydrogen peroxide; SA: salicylic acid. Degree of freedom (Df) = 3 Significant effect is indicated in boldface as follows: *** *p* ≤ 0.001.

## Data Availability

All data are available in this study.

## Supplementary Materials

The following are available online. Figure S1: Correlational analysis among flavonoid biosynthesis genes and total phenolics and total flavonoids content; Table S1: Sequences of primers for the amplification of genes in flavonoid biosynthesis pathway.

## Sample Availability

Samples of the compounds are not available from the authors.

## References

[1] Shafroth P.B., Stromberg J.C., Patten D.T. (2000). Woody riparian vegetation response to different alluvial water table regimes. West. N. Am. Nat..

[2] Ma D., Sun D., Wang C., Li Y., Guo T. (2014). Expression of flavonoid biosynthesis genes and accumulation of flavonoid in wheat leaves in response to drought stress. Plant Physiol. Biochem..

[3] Li C., Wang K. (2003). Differences in drought responses of three contrasting *Eucalyptus microtheca* F. Muell. populations. For. Ecol. Manag..

[4] Yin C., Wang X., Duana B., Luob J., Li C. (2005). Early growth, dry matter allocation and water use efficiency of two sympatric *Populus* species as affected by water stress. Environ. Exp. Bot..

[5] Zhang X., Wu N., Li C. (2005). Physiological and growth responses of *Populus davidiana* ecotypes to different soil water contents. J. Arid Environ..

[6] Salem N., Msaada K., Dhifi W., Limam F., Marzouk B. (2014). Effect of salinity on plant growth and biological activities of *Carthamus tinctorius* L. extracts at two flowering stages. Acta Physiol. Plant.

[7] Pirbalouti A.G., Malekpoor F., Salimi A., Golparvar A. (2017). Exogenous application of chitosan on biochemical and physiological characteristics, phenolic content and antioxidant activity of two species of basil (*Ocimum ciliatum* and *Ocimum basilicum*) under reduced irrigation. Sci. Hortic..

[8] Zhang W., Cao Z., Xie Z., Lang D., Zhou L., Chu Y., Zhao Q., Zhang X., Zhao Y. (2017). Effect of water stress on roots biomass and secondary metabolites in the medicinal plant *Stellaria dichotoma* L. var. lanceolata Bge. Sci. Hortic..

[9] Kozlowski T.T., Pallardy S. (2002). Acclimation and adaptive responses of woody plants to environmetal stresses. Bot. Rev..

[10] Ruiz-Sánchez M.C., Domingo R., Pérez-Pastor A. (2007). Daily variations in water relations of apricot trees under different irrigation regimes. Biol. Plant.

[11] Shvaleva A.L., Costa E., Silva F., Breia E., Jouve J., Hausman J.F., Almeida M.H., Maroco J.P., Rodrigues M.L., Pereira J.S. (2006). Metabolic responses to water deficit in two *Eucalyptus globulus* clones with contrasting drought sensitivity. Tree Physiol..

[12] Horváth E., Pál M., Szalai G., Páldi E., Janda T. (2007). Exogenous 4-hydroxybenzoic acid and salicylic acid modulate the effect of short-term drought and freezing stress on wheat plants. Biol. Plant.

[13] Ren J., Dai W., Xuan Z., Yao Y., Korpelainen H., Li C. (2007). The effect of drought and enhanced UV-B radiation on the growth and physiological traits of two contrasting poplar species. For. Ecol. Manag..

[14] Wang T., Zhang X., Li C. (2007). Growth, abscisic acid content, and carbon isotope composition in wheat cultivars grown under different soil moisture. Biol. Plant.

[15] Tardieu F., Tuberosa R. (2010). Dissection and modeling of abiotic tolerance plants. Curr. Opin. Plant Biol..

[16] Valliyodan B., Nguyen H.T. (2006). Understanding regulatory networks and engineering for enhanced drought tolerance in plants. Curr. Opin. Plant Biol..

[17] Rosa D.D., Furtado E.L., Boava L.P., Marino C.L., Mori E.S., Guerrini I.A., Veline E.D., Wilcken C.F. (2010). *Eucalyptus* ESTs involved in mechanisms against plant pathogens and environmental stresses. Summa Phytopathol..

[18] Daayf F., El-Hadrami A., El-Bebany A.F., Henriquez M.A., Yao Z., Derksen H., El-Hadrami I., Adam L.R. (2012). Phenolic Compounds in Plant Defense and Pathogen Counter-Defense Mechanisms. Recent Advances in Polyphenol Research.

[19] Ferreyra M.L.F., Rius S.P., Casati P. (2012). Flavonoids: Biosynthesis, biological functions, and biotechnological applications. Front. Plant Sci..

[20] Treutter D. (2006). Significance of flavonoids in plant resistance: A review. Environ. Chem. Lett..

[21] Shih C.H., Chu H., Tang L.K., Sakamoto W., Maekawa M., Chu I.K., Wang M., Lo C. (2008). Functional characterization of key structural genes in rice flavonoid biosynthesis. Planta.

[22] Constabel C.P., Lindroth R., Jansson S., Bhalerao R., Groover A. (2010). The Impact of Genomics on Advances in Herbivore Defense and Secondary Metabolism in *Populus*. Genetics and Genomics of Populus.

[23] Daniels W., Rautenbach F., Marnewick J.L., Valentine A.J., Babajide O.J., Mabusela W.T. (2015). Environmental stress effect on the phytochemistry and antioxidant activity of a South African bulbous geophyte, *Gethyllis multifolia* L. Bolus. S. Afr. J. Bot..

[24] Bistgani Z.E., Siadat S.A., Bakhshandeh A., Pirbalouti A.G., Hashemi M. (2017). Morpho-physiological and phytochemical traits of (*Thymus daenensis* Celak.) in response to deficit irrigation and chitosan application. Acta Physiol. Plant.

[25] Vosoughi N., Gomarian M., Pirbalouti A.G., Khaghani S., Malekpoor F. (2018). Essential oil composition and total phenolic, flavonoid contents, and antioxidant activity of sage (*Salvia officinalis* L.) extract under chitosan application and irrigation frequencies. Ind. Crops Prod..

[26] Markham K.R., Tanner G.J., Caasi-Lit M., Whitecross M.I., Nayudu M., Mitchell K.A. (1998). Possible protective role for 3′, 4′-dihydroxyflavones induced by enhanced UV-B in a UV-tolerant rice cultivar. Phytochemistry.

[27] Khoyerdi F.F., Shamshiri M.H., Estaji A. (2016). Changes in some physiological and osmotic parameters of several pistachio genotypes under drought stress. Sci. Hortic..

[28] Gharibi S., Tabatabaei B.E., Saeidi G., Goli S.A. (2016). Effect of Drought Stress on Total Phenolic, Lipid Peroxidation, and Antioxidant Activity of Achillea Species. Appl. Biochem. Biotechnol..

[29] Farag M.A., Deavours B.E., de Fáltima Â., Naoumkina M., Dixon R.A., Sumner L.W. (2009). Integrated metabolite and transcript profiling identify a biosynthetic mechanism for hispidol in *Medicago truncatula* cell cultures. Plant Physiol..

[30] Hall R., Heybroek H. (1997). Biology of *Populus* and its implications for management and conservation. For. Sci..

[31] Brunner A.M., Busov V.B., Strauss S.H. (2004). Poplar genome sequence: Functional genomics in an ecologically dominant plant species. Trends Plant Sci..

[32] Tuskan G.A., Difazio S., Jansson S., Bohlmann J., Grigoriev I., Hellsten U., Putnam N., Ralph S., Rombauts S., Salamov A. (2006). The genome of black cottonwood, *Populus trichocarpa* (Torr. & Gray). Science.

[33] Monclus R., Dreyer E., Villar M., Delmotte F.M., Delay D., Petit J., Barbaroux C., Le Thiec D., Bréchet C., Brignolas F. (2006). Impact of drought on productivity and water use efficiency in 29 genotypes of *Populus deltoides* × *Populus nigra*. New Phytol..

[34] Yin C., Duan B., Wang X., Li C. (2004). Morphological and physiological responses of two contrasting Poplar species to drought stress and exogenous abscisic acid application. Plant Sci..

[35] Taylor G. (2002). *Populus*: Arabidopsis for forestry. Do we need a model tree?. Ann. Bot..

[36] Mader M., Le Paslier M.C., Bounon R., Berard A., Rampant P.F., Fladung M., Leple J.C., Kersten B. (2016). Whole-genome draft assembly of *Populus tremula* × *P. alba* clone INRA 717-1B4. Silvae Genet..

[37] Bai Q., Duan B., Ma J., Fen Y., Sun S., Long Q., Lv J., Wan D. (2020). Coexpression of *PalbHLH1* and *PalMYB90* genes from *Populus alba* enhances pathogen resistance in poplar by increasing the flavonoid content. Front. Plant Sci..

[38] Wu Q., Chen M., Zhou H., Zhou X., Wang Y. (2015). Metabolite profiles of *Populus* in response to pathogen stress. Biochem. Biophys. Res. Commun..

[39] Syvertsen J.P., Garcia-Sanchez F. (2014). Multiple abiotic stresses occurring with salinity stress in citrus. Environ. Exp. Bot..

[40] Sarker U., Oba S. (2018). Augmentation of leaf color parameters, pigments, vitamins, phenolic acids, flavonoids and antioxidant activity in selected *Amaranthus tricolor* under salinity stress. Sci. Rep..

[41] Yu J.J., Jin X., Sun X.M., Gao T.X., Chen X.M., She Y.M., Jiang T.B., Chen S.X., Dai S.J. (2017). Hydrogen peroxide response in leaves of Poplar (*Populus simonii* × *Populus nigra*) revealed from physiological and proteomic analyses. Int. J. Mol. Sci..

[42] Roby G., Harbertson J.F., Adams D.A., Matthews M.A. (2004). Berry size and vine water deficits as factors in winegrape composition: Anthocyanins and tannins. Aust. J. Grape Wine Res..

[43] Lenka S.K., Katiyar A., Chinnusamy V., Bansal K.C. (2011). Comparative analysis of drought-responsive transcriptome in Indica rice genotypes with contrasting drought tolerance. Plant Biotechnol. J..

[44] Vasquez-Robinet C., Mane S.P., Ulanov A.V., Watkinson J.I., Stromberg V.K., De Koeyer D., Schafleitner R., Willmot D.B., Bonierbale M., Bohnert H.J. (2008). Physiological and molecular adaptations to drought in Andean potato genotypes. J. Exp. Bot..

[45] Huang J., Gu M., Lai Z., Fan B., Shi K., Zhou Y., Yu J., Chen Z. (2010). Functional analysis of the Arabidopsis PAL gene family in plant growth, development, and response to environmental stress. Plant Physiol..

[46] Dempsey D.M.A., Vlot A.C., Wildermuth M.C., Klessig D.F. (2011). Salicylic acid biosynthesis and metabolism. Arab. Book.

[47] Liu M.L., Li X.R., Liu Y.B., Cao B. (2013). Regulation of *flavanone 3-hydroxylase* gene involved in the flavonoid biosynthesis pathway in response to UV-B radiation and drought stress in the desert plant, *Reaumuria soongorica*. Plant Physiol. Biochem..

[48] Castellarin S.D., Matthews M.A., Di Gaspero G., Gambetta G.A. (2007). Water deficits accelerate ripening and induce changes in gene expression regulating flavonoid biosynthesis in grape berries. Planta.

[49] Kim B.G., Lee E.R., Ahn J.H. (2012). Analysis of flavonoid contents and expression of flavonoid biosynthetic genes in *Populus euramericana* Guinier in response to abiotic stress. J. Korean Soc. Appl. Biol. Chem..

[50] Arbona V., Manzi M., Ollas C., Gomez-Cadenas A. (2013). Metabolomics as a tool to investigate abiotic stress tolerance in plants. Int. J. Mol. Sci..

[51] Fraser C.M., Chapple C. (2011). The phenylpropanoid pathway in Arabidopsis. Arab. Book.

[52] Hussain S., Rao M.J., Anjum M.A., Ejaz S., Zakir I., Ali M.A., Ahmad N., Ahmad S., Hasanuzzaman M., Hakeem K.R., Nahar K., Alharby H.F. (2019). Oxidative Stress and Antioxidant defense in plants under drought conditions. Plant Abiotic Stress Tolerance: Agronomic, Molecular and Biotechnological Approaches.

[53] Laxa M., Liebthal M., Telman W., Chibani K., Dietz K.-J. (2019). The role of the plant antioxidant system in drought tolerance. Antioxidants.

[54] Rao M.J., Xu Y., Huang Y., Tang X., Deng X., Xu Q. (2019). Ectopic expression of citrus *UDP-GLUCOSYL TRANSFERASE* gene enhances anthocyanin and proanthocyanidins contents and confers high light tolerance in Arabidopsis. BMC Plant Biol..

[55] Hussain S., Khalid M.F., Saqib M., Ahmad S., Zafar W., Rao M.J., Morillon R., Anjum M.A. (2018). Drought tolerance in citrus rootstocks is associated with better antioxidant defense mechanism. Acta Physiol. Plant.

[56] Okello O.P., Nawiri M.P., Musila W., Gweyi-Onyango J.P. (2017). Water stress effect on total antioxiant activity and total phenolic content of *Solanum scabrum* and *Solanum scabrum* in Kiambu, Kenya. Int. J. Biochem. Res. Rev..

[57] Alothman M., Bhat R., Karim A. (2009). Antioxidant capacity and phenolic content of selected tropical fruits from Malaysia, extracted with different solvents. Food Chem..

[58] Miao J., Li X., Zhao C., Gao X., Wang Y., Gao W. (2018). Active compounds, antioxidant activity and α -glucosidase inhibitory activity of different varieties of Chaenomeles fruits. Food Chem..

[59] Abdallah S.B., Rabhi M., Harbaoui F., Zar-kalai F., Lachâal M., Karray-Bouraoui N. (2013). Distribution of phenolic compounds and antioxidant activity between young and old leaves of *Carthamus tinctorius* L. and their induction by salt stress. Acta Physiol. Plant.

[60] Hudz N., Ivanova R., Brindza J., Grygorieva O., Schubertová Z., Ivanišová E. (2017). Approaches to the determination of antioxidant activity of extracts from bee bread and safflower leaves and flowers. Potravin. Slovak J. Food Sci..

[61] Salem N., Msaada K., Hamdaoui G., Limam F., Marzouk B. (2011). Variation in phenolic composition and antioxidant activity during flower development of safflower (*Carthamus tinctorius* L.). J. Agric. Food Chem..

[62] Ramel F., Birtic S., Ginies C., Soubigou-Taconnat L., Triantaphylides C., Havaux M. (2012). Carotenoid oxidation products are stress signals that mediate gene responses to singlet oxygen in plants. Proc. Natl. Acad. Sci. USA.

[63] Hou X., Rivers J., León P., McQuinn R.P., Pogson B.J. (2016). Synthesis and function of apocarotenoid signals in plants. Trends Plant Sci..

[64] Yu J., Chen S., Zhao Q., Wang T., Yang C., Diaz C., Sun G., Dai S. (2011). Physiological and proteomic analysis of salinity tolerance in *Puccinellia tenuiflora*. J. Proteome Res..

[65] Sumanta N., Haque C.I., Nishika J., Suprakash R. (2014). Spectrophotometric analysis of chlorophylls and carotenoids from commonly grown fern species by using various extracting solvents spectrophotometric analysis of chlorophylls and carotenoids from commonly grown fern species by using various extracting solvents. Res. J. Chem. Sci..

[66] Pfaffl M.W., Horgan G.W., Dempfle L. (2002). Relative expression software tool (REST) for group-wise comparison and statistical analysis of relative expression results in real-time PCR. Nucleic Acids Res..

[67] Tian D., Liu Y., Tian L., Wan M., Zheng B., Shi X. (2019). Involvement of *Populus* CLEL peptides in root development. Tree Physiol..

[68] Velioglu Y.S., Mazza G., Gao L., Oomah B.D. (1998). Antioxidant activity and total phenolics in selected fruits, vegetables, and grain products. J. Agric. Food Chem..

[69] Dewanto V., Wu X., Adom K.K., Liu R.H. (2002). Thermal processing enhances the nutritional value of tomatoes by increasing total antioxidant activity. J. Agric. Food Chem..

[70] Broadhurst R.B., Jones W.T. (1978). Analysis of condensed tannins using acidified vanillin. J. Sci. Food Agric..

[71] Nakata M., Ohme-Takagi M. (2014). Quantification of anthocyanin content. Bio-Protocol.

[72] Özgen M., Scheerens J.C., Neil Reese R., Miller R.A. (2010). Total phenolic, anthocyanin contents and antioxidant capacity of selected elderberry (*Sambucus canadensis* L.) accessions. Pharm. Mag..

[73] Velikova V., Yordanov I., Edreva A. (2000). Oxidative stress and some antioxidant systems in acid rain-treated bean plants: Protective role of exogenous polyamines. Plant Sci..

[74] Feng Y., Zhang M., Guo Q., Wang G., Gong J., Xu Y., Wang W. (2014). Manipulation of monoubiquitin improves chilling tolerance in transgenic tobacco (*Nicotiana tabacum*). Plant Physiol. Biochem..

[75] Warrier R., Paul M., Vineetha M. (2013). Estimation of salicylic acid in *Eucalyptus* leaves using spectrophotometric methods. Genet. Plant Physiol..

