# Supplementary material for: Expression Profiling of Flavonoid Biosynthesis Genes and Secondary Metabolites Accumulation in Populus under Drought Stress
Source: Molecules. 2021 Sep 13;26(18):5546. doi: 10.3390/molecules26185546 (PMC8467073; doi:10.3390/molecules26185546)
Supplement: Supplementary file 1 [file molecules-26-05546-s001.zip › molecules-1366216-supplementary.pdf]

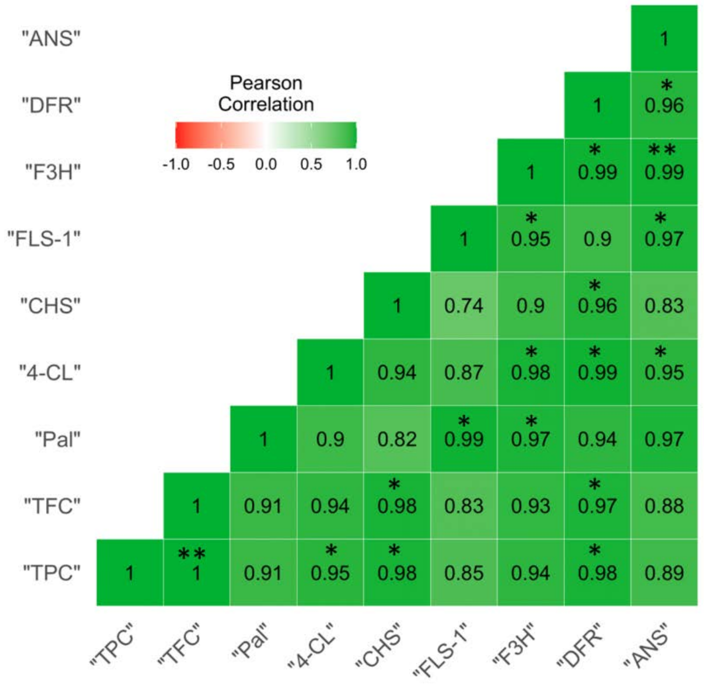

Figure S1. Correlational analysis among flavonoid biosynthesis genes and total phenolics and total flavonoids content.

Table S1. Sequences of primers for the amplification of genes in flavonoid biosynthesis pathway

Gene	Forward Primer (5′ → 3′)	Reverse Primer (5′ → 3′)
PAL	CCAGGCTCCTCAATAACAACAT	CAAAGAATCCAGATTCAATGCC
4-CL	GATGTTAAGGTCATGTGCGTAGAC	CTTTTGGTAACCCTGTAGTCCCT
CHS	GAGACAACCTGCGGTAGTGGA	TTGCTGGTCGAACGATCTTATT
F3H	GACCCGAAGGCAGATGAA	CACTCCAAAGCAGGAACAAA
FLS-1	ATCGAACCACGGTGGCTAA	GTCTTGAACTTTGGAGGATTGTCT
DFR	GCTGGAGTGATTTGGACTTTGT	CAGGAGACTTGGTGGCATTG
ANS	CAACTACTACCCCAAGTGCCC	GCTGTGAGCTTCAACCCCTAA
Act	TCATCGGAATGGAAGCTGCTGGTA	TAGTGGAACCACCACTGAGCACAA
